# Radiomics Analysis of Dynamic Contrast-Enhanced Magnetic Resonance Imaging for the Prediction of Sentinel Lymph Node Metastasis in Breast Cancer

**DOI:** 10.3389/fonc.2019.00980

**Published:** 2019-09-30

**Authors:** Jia Liu, Dong Sun, Linli Chen, Zheng Fang, Weixiang Song, Dajing Guo, Tiangen Ni, Chuan Liu, Lin Feng, Yuwei Xia, Xiong Zhang, Chuanming Li

**Affiliations:** ^1^Department of Radiology, The Second Affiliated Hospital of Chongqing Medical University, Chongqing, China; ^2^Department of Breast and Thyroid Surgery, The Second Affiliated Hospital of Chongqing Medical University, Chongqing, China; ^3^Department of Radiology, Affiliated Hospital of Chuanbei Medical College, Nanchong, China; ^4^Huiying Medical Technology, Beijing, China

**Keywords:** breast cancer, DCE-MRI, radiomics, sentinel lymph node metastasis, automatic machine learning

## Abstract

**Purpose:** To investigate whether a combination of radiomics and automatic machine learning applied to dynamic contrast-enhanced magnetic resonance imaging (DCE-MRI) of primary breast cancer can non-invasively predict axillary sentinel lymph node (SLN) metastasis.

**Methods:** 62 patients who received a DCE-MRI breast scan were enrolled. Tumor resection and sentinel lymph node (SLN) biopsy were performed within 1 week after the DCE-MRI examination. According to the time signal intensity curve, the volumes of interest (VOIs) were delineated on the whole tumor in the images with the strongest enhanced phase. Datasets were randomly divided into two sets including a training set (~80%) and a validation set (~20%). A total of 1,409 quantitative imaging features were extracted from each VOI. The select K best and least absolute shrinkage and selection operator (Lasso) were used to obtain the optimal features. Three classification models based on the logistic regression (LR), XGboost, and support vector machine (SVM) classifiers were constructed. Receiver Operating Curve (ROC) analysis was used to analyze the prediction performance of the models. Both feature selection and models construction were firstly performed in the training set, then were further tested in the validation set by the same thresholds.

**Results:** There is no significant difference between all clinical and pathological variables in breast cancer patients with and without SLN metastasis (*P* > 0.05), except histological grade (*P* = 0.03). Six features were obtained as optimal features for models construction. In the validation set, with respect to the accuracy and MSE, the SVM demonstrated the highest performance, with an accuracy, AUC, sensitivity (for positive SLN), specificity (for positive SLN) and Mean Squared Error (MSE) of 0.85, 0.83, 0.71, 1, 0.26, respectively.

**Conclusions:** We demonstrated the feasibility of combining artificial intelligence and radiomics from DCE-MRI of primary tumors to predict axillary SLN metastasis in breast cancer. This non-invasive approach could be very promising in application.

## Introduction

Breast cancer is a major disease that seriously threatens women's physical health and quality of life. In recent years, the incidence rate of breast cancer has been increasing, with an estimated 1.7 million cases annually (1). With the development of standardization and precise treatment, requirements for the postoperative quality of life of breast cancer patients have also increased. Axillary lymph nodes (ALNs) receive approximately 70% of the lymphatic drainage of the breast and are the most important lymphatic metastatic pathways for breast cancer. The status of ALNs is of great significance for judging the clinical stage of breast cancer, selecting a treatment plan, and evaluating the prognosis (2). The ALN status also plays an important role in adjuvant treatment plan selection after surgery. The sentinel lymph node (SLN) in breast cancer is the lymph node that is the closest to the tumor in the direction of lymphatic drainage, the first to receive lymphatic drainage and the earliest to metastasize. The SLN status is used to predict the involvement of additional ALNs and is an important indicator to guide the clinical need for ALN dissection (ALND) (3). Histopathological examination after SLN biopsy (SLNB) is the gold standard for the evaluation of SLN metastasis. However, SLNB is an invasive operation, and patients are at risk of lymphedema, decreased muscle strength, and sensory disturbance as the result of the operation (4). If SLN metastasis can be predicted with a non-invasive method before surgery, the complications caused by SLNB can be avoided, and the quality of the patient's life can be greatly improved.

As a non-invasive method, medical imaging technology has shown great potential in breast cancer detection and ALN status assessment. Among the existing breast imaging modalities, dynamic contrast-enhanced magnetic resonance imaging (DCE-MRI) is considered the best tool for evaluating the extent of the tumor and tumor heterogeneity by analyzing the patterns of enhancement (5–7). For ALN metastasis staging, previous reports have primarily focused on the node size, cortical thickness, disappearance of lymph parenchyma, diffusion-weighted imaging (DWI) signals, and enhancement mode. However, this information is far from sufficient for the prediction of SLN metastasis. In addition, due to the limitations of subjective factors such as the experience and knowledge level of clinicians, the diagnosis of early SLN metastasis through MRI is still not ideal.

Recently, growing attention has been focused on discovering and using the quantitative image features of the original MRI, with the emergence of “radiomics.” Radiomics was developed by the Dutch scholar Philippe Lambin in 2012. This approach utilizes an automated high-throughput extraction of a vast (200+) number of quantitative features from original MRI images, excavating latent data that are not visually discernible (8–10). This method includes several processes, such as image collection, lesion segmentation, feature extraction and screening, and model construction. This technology greatly expanded the guiding value of medical imaging in clinical practice. Radiomics has been applied in the diagnosis and prognostic evaluation of lung cancer, prostate cancer, liver cancer and breast cancer (11–14). Specially, the radiomic features of primary tumors have shown a close correlation to lymph node metastasis. The radiomic features of primary colorectal cancer have been reported to successfully predict lymph node metastasis prior to surgery (15). In this study, we investigate whether the combination of radiomics and the automatic machine learning of original DCE-MRI images can predict SLN metastasis before biopsy.

## Methods

### Patients and Data Management

This study was approved by the Medical Ethics Committee of our hospital, and all patients provided written consent for the study. A total of 85 patients with histologically confirmed breast cancer from March 2013 to December 2018 were enrolled. The inclusion criteria were as follows: (1) patients had breast cancer confirmed by pathology; (2) patients underwent a DCE-MRI scan before tumor resection or biopsy; (3) patients received tumor resection and SLNB within 1 week after MRI examination. The exclusion criteria included the following: MRI examination data were incomplete, or image quality was poor. Ultimately, a total of 62 patients were included in this study (62 lesions containing 35 SLN metastasis and 27 non-SLN metastasis). The details of the clinical and histopathological characteristics are shown in Table 1.

**Table 1:** Clinical and histopathological characteristics.

**Group**	**Patients with positive SLN (*n* = 35)**	**Patients with negative SLN (*n* = 27)**	***P*-value**
Number of lesions	35	27	
Mean age (mean ± SD)	48.14 ± 8.35	49.78 ± 12.53	0.541
Mean size (mean ± SD)	3.60 ± 1.85	2.98 ± 1.45	0.157
**Histological type**			
Invasive ductal carcinoma	35	27	
**Histological grade**			0.03
I	6	14	
II	11	9	
III	18	4	
**ER**			0.697
+	23	19	
–	12	8	
**PR**			0.812
+	21	17	
–	14	10	
**HER-2**			0.780
+	27	20	
–	8	7	
**Ki-67**			0.094
+	23	12	
–	12	15	

*Two-tailed two-sample t-test with unequal variances was used to compare the age and the primary tumor size. Chi-square cross-tabulation was used to test the histological grade and immunohistochemical marker (ER, PR, HER2, and Ki-67). SLN, sentinel lymph node; SD, standard deviation*.

MR imaging was performed on a 3.0-T MR scanner (Discovery MR750, GE Medical Systems, Milwaukee, WI, USA) equipped with a breast coil in the prone position by using the following sequences: T2 weighted fat-suppressed axial-, T2 weighted fat-suppressed sagittal-, T1 weighted fat-saturated axial-, T1 weighted fat-suppressed axial-series in dynamic phase and diffusion imaging. Images from a T1 weighted fat-suppressed dynamic sequence using a 3D fast gradient echo sequence (VIBRANT 3D, TR = 4.5, TE = 2.1; Flip = 10°, Matrix 384 × 256, NEX = 1, Fov = 34 cm, layer thickness = 1.2 mm, interval = 0 mm) were used in analysis. The first frame was acquired before the injection of the contrast agent (Gd-DTPA, 0.1 mmol/kg body) into the left elbow vein. The second phase began after 40 s with contrast injection, and five phases were then scanned continuously. Each phase was acquired over 58 s, and the total scan time was 6 min and 46 s.

### SLNB and Pathologic Assessment

SLNB was performed for all patients within 1 week after MRI examination. Methylene blue tracer was used to identify the SLN during operation. SLN metastasis was confirmed by final histopathology. The SLN was defined as metastatic when there were macrometastases (>2 mm) or micrometastases (0.2–2 mm or deposits >200 cells). The results were confirmed by two pathologists with 10 and 12 years of experience.

Histopathology of the initial breast tumor was performed after primary tumor resection, and the expression levels of estrogen receptor (ER), progesterone receptor (PR), human epidermal growth factor receptor-2 (HER2), and proliferation marker Ki-67 in each breast cancer patient were determined using streptavidin-peroxidase (SP) immunohistochemistry (IHC). The ER or PR status was considered to be positive when at least 1% of the tumor cell nuclei showed staining for ER or PR. The HER2 status was determined to be positive when the IHC staining intensity score was ≥3. An IHC HER2 score of 2+ was considered with confirmation of gene amplification by fluorescence *in situ* hybridization (FISH). A sample was considered positive if the Ki-67 level was >14% and was otherwise considered negative.

### Radiomics Workflow

**Figure 1:**
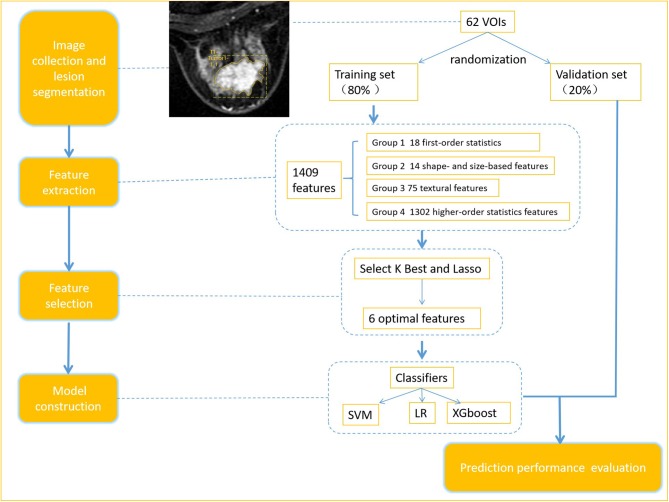
Radiomics workflow.

The radiomics workflow is presented in Figure 1, including (1) image collection, (2) lesion segmentation and radiomic feature extraction, (3) features selection and models construction (in training set), and (4) prediction performance evaluation.

### Image Segmentation and Radiomic Feature Extraction

Axial DCE-MRI Digital Imaging and Communications in Medicine (DICOM) images were archived from the Picture Archiving and Communication System (PACS). Time signal intensity curves for tumor lesions in the DCE-MRI images were calculated using a GE Advanced Workstation ADW4.4. Based on these curves, the volumes of interest (VOIs) were delineated on the whole tumor in the images with the strongest enhanced phase. The VOIs were determined manually by a radiologist with 10 years of experience who was blinded to the clinical information of the patients, and all contours were reviewed by another senior radiologist with 20 years of experience. If the discrepancy was ≥5%, the senior radiologist determined the tumor borders (9). Cohen's kappa method was used to assess inter-reader agreement. A total of 62 VOIs were manually determined from 62 patient images.

A total of 1,409 quantitative imaging features were automatically extracted from each VOI; these features were categorized into four groups. Group 1 (first order statistics) consisted of 18 descriptors that quantitatively delineate the distribution of voxel intensities within the MR image through commonly used and basic metrics. Group 2 (shape- and size-based features) contained 14 three-dimensional features that reflect the shape and size of the region. Calculated from Gray Level Co-occurence Matrix (GLCM), Gray Level Run Length Matrix (GLRLM), Gray Level Size Zone Matrix (GLSZM), Gray Level Difference Matrix (GLDM) and Neighborhood Gray-Tone Difference Matrices (NGTDM) 75 textural features that can quantify region heterogeneity differences were classified into group 3 (texture features). Finally, group 4 (higher order statistics features) included the intensity and texture features derived from filters transformation of the original image, we used seven types of filters: exponential, square, square root, logarithm, gradient, lbp-2D and wavelet (wavelet-LLL, wavelet-HHH, wavelet-HLL, wavelet-HHL, wavelet-LLH, wavelet-HLH, wavelet-LHL, wavelet-LHH).

### Feature Selection

Computer-generated random datasets were used to assign 80% of datasets to the training set (49 patients with 27 positive SLN) and 20% of datasets to the validation set (13 patients with 7 positive SLN).To reduce the dimensionality of the features, Select K best and the least absolute shrinkage and selection operator (Lasso) algorithm methods were used to obtain the optimal features from the training set in Radcloud platform (Huiying Medical Technology Co., Ltd). Select K Best can select features based on relevance, retaining the k highest scores, based on the select K best findings. The features that did not show statistical differences (*P* > 0.05) were removed. This process can be viewed as a preconditioning of the predictive model. The purpose of this method was to minimize the Lasso cost function and to obtain all features with non-zero coefficients. The minimized objective function is:

$$\begin{array}{l} \underset{w}{\min}\frac{1}{2\text{n}}||Xw-y|{|}_{2}^{2}+\alpha {\left||w|\right|}_{1} \end{array}$$

where *X* is a matrix of radiomic features, *y* is a vector of sample labels, *n* is the number of samples, *w* is a coefficient vector of the regression model, and α||*w*||_1_ is the Lasso penalty with the constant α and the ℓ_1_-norm of the coefficient vector ||*w*||_1_ (15, 16).

Data processing was performed as follows. First, all radiomic features were standardized using the StandardScaler function by removing the mean and dividing by its standard deviation, and each set of feature values was converted to a mean of 0 with a variance of 1. Then, a 10-fold cross-validation was performed based on standardized features, and the optimal α parameter was obtained from the minimum of the average mean square error. Finally, the Lasso function was used to select the relevant features based on the optimal α parameters, and the coefficients were calculated for each feature; then, radiomic features with non-zero coefficients were obtained.

### Performance of the Radiomics Signature

The predictive performance of models was assessed in the validation set by the same thresholds determined in the training set.

### Statistical Analysis

Two-tailed two-sample *t*-test with unequal variances was used to compare the age and the primary tumor size of the patients with and without SLN metastasis. Chi-square cross-tabulation was used to compare the histological grade and immunohistochemical markers (ER, PR, HER2, and Ki-67) levels. Statistical Package for Social Sciences (SPSS) software version 23.0 (SPSS Inc., Chicago, IL, USA) was used. *P*-values < 0.05 were considered statistically significant.

Classification models based on logistic regression (LR), support vector machine (SVM), and XGboost were constructed using Radcloud platform (Huiying Medical Technology Co., Ltd). The Receiver Operating Curve (ROC) analysis was used to illustrate the prediction performance. The AUC, accuracy, sensitivity, specificity and Mean Squared Error (MSE) were calculated.

## Results

### Clinical and Histopathological Characteristics

The result of clinical and histopathological characteristics were shown in the Table 1. All the clinical and pathological variables between the patients with and without SLN metastasis had no significant differences (*P* > 0.05), except histological grade (*P* = 0.03).

### Feature Extraction and Selection

**Figure 2:**
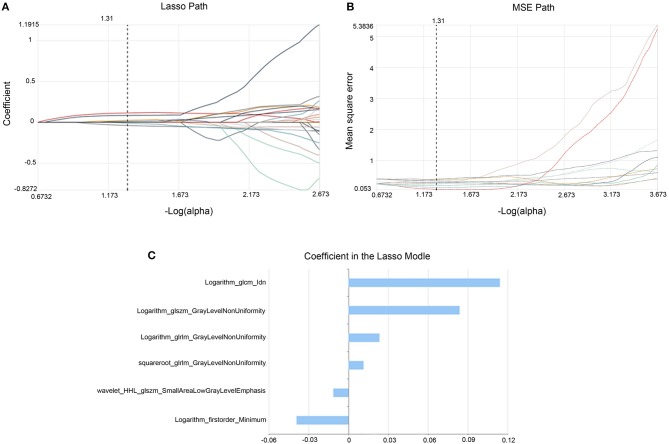
Lasso algorithm for feature selection. The Lasso path **(A)** showed coefficient profiles along the full path of possible values for radiomic features. The optimal α value of 0.27 with -log(*a*) = 1.31 was selected. The MSE path **(B)** showed that the dotted vertical line was plotted at the value selected using 10-fold cross-validation in **(A)**. The coefficients in the Lasso model **(C)** resulted in 6 features corresponding to the selected optimal values.

In the training set 35 features out of 1,409 features were selected using select K best method. Then six optimal features were obtained with the Lasso methods (Figure 2), including six higher-order statistics features (Table 2).

**Table 2:** Description of the selected radiomic features and their associated feature types and filters.

**Radiomic features**	**Types**	**Associated filters**
Idn	glcm	Logarithm
GrayLevelNonUniformity	glszm	Logarithm
GrayLevelNonUniformity	glrlm	Logarithm
Minimum	first order statistics	Logarithm
GrayLevelNonUniformity	glrlm	Squareroot
SmallAreaLowGrayLevelEmphasis	glszm	Wavelet-HHL

*glcm, level cooccurrence matrix; glrlm, gray level run length matrix; glszm, gray-level size zone matrix*.

### Prediction Performance of Classification Models

**Figure 3:**
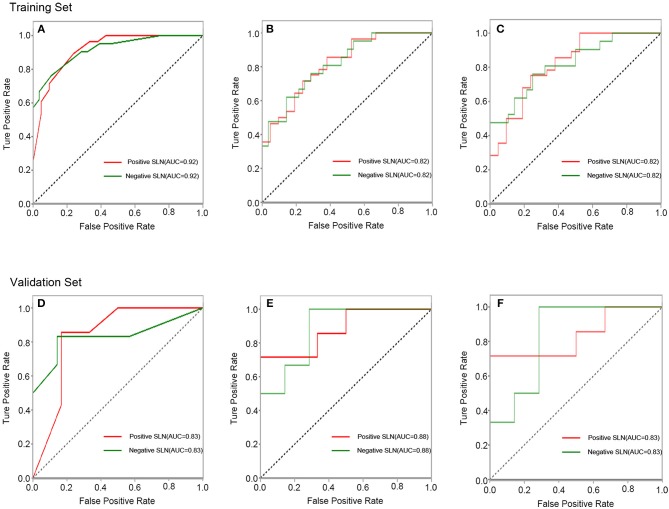
ROC curves of the XGboost **(A)**, LR **(B)**, and SVM **(C)** classifiers in training set. ROC curves of the XGboost **(D)**, LR **(E)**, and SVM **(F)** classifiers in validation set.

In the training set, the accuracy, sensitivity (for positive SLN), specificity (for positive SLN) and AUC of SVM, LR and GXboost were 0.76, 0.75, 0.76, 0.82; 0.71, 0.71, 0.71, 0.82; 0.84, 0.89, 0.76, 0.92, respectively. In the validation set, the overall accuracy, sensitivity (for positive SLN), specificity (for positive SLN) and AUC of SVM, LR and XGboost were 0.85, 0.71, 1, 0.83; 0.77, 0.71, 0.83, 0.88; 0.85, 0.86, 0.83, 0.83, respectively. MSE of SVM, LR, and XGboost were 0.20, 0.19, 0.17 in the training set and 0.26, 0.28, 0.34 in the validation set (Figure 3, Table 3). With regard to accuracy and MSE, the SVM demonstrated the best performance among the three models.

**Table 3:** The results of radiomic analysis for classifications.

**Classifiers**	**SLN metastasis**	**Training set**					**Validation set**				
		**ACC**	**SEN**	**SPE**	**AUC**	**MSE**	**ACC**	**SEN**	**SPE**	**AUC**	**MSE**
SVM	Positive	0.76	0.75	0.76	0.82	0.20	0.85	0.71	1	0.83	0.26
	Negative		0.76	0.75				1	0.71		
XGboost	Positive	0.84	0.89	0.76	0.92	0.17	0.85	0.86	0.83	0.83	0.34
	Negative		0.76	0.89				0.83	0.86		
LR	Positive	0.71	0.71	0.71	0.82	0.20	0.77	0.71	0.83	0.88	0.28
	Negative		0.71	0.71				0.83	0.71		

*SVM, support vector machine; LR, logistic regression; ACC, accuracy; SEN, sensitivity; SPE, specificity; AUC, area under the curve; MSE, mean squared error; SLN, sentinel lymph node*.

## Discussion

The accurate detection of ALN metastases in breast cancer is critical for surgical planning, adjuvant therapy planning, and prognostication. The determination of a negative ALN status can eliminate the need for ALN dissection. Until now, SLNB and pathological biopsy have been the most popular methods for determining ALN status. However, the side effects of SLNB should not be neglected. For example, lymphedema is reported in 5–8% of patients after a SLNB (17, 18). Other common complications include pain, paresthesia, decreased arm strength and shoulder stiffness (19). Therefore, in this study, we attempted to establish a new non-invasive method with high accuracy to prevent node-negative patients from undergoing unnecessary invasive staging procedures.

Radiomics is a relatively new technique (9) that can be used to characterize intratumor heterogeneity and to improve diagnostic and predictive accuracy. This approach utilizes an automated high-throughput extraction of a vast number of quantitative features from medical images, excavating latent data that are not visually discernible. This method is different from traditional practice in which images are subjected to only visual interpretation (10, 20). In this study, we aimed to predict SLN metastasis by selecting an optimal artificial intelligence model based on the radiomic features of primary tumors. Compared with the LR and XGboot classifiers, we found that the SVM results based on the strongest enhanced DCE-MRI images gave the best classification efficacy, with accuracy (0.85) and MSE (0.26) in the validation set. Our results suggest that the SVM and radiomics can be combined as a new approach for predicting ALN metastasis, which can guide further treatment planning. This method can prevent unnecessary invasive SLNB and its associated complications. Thus, this approach is a valuable means to help clinicians determine the appropriate treatment for their patients, with wide applicability in clinical practice. Previously, other studies have reported the clinical application of radiomics predicting lymph node metastasis in bladder and lung cancer. Wu et al. (21) demonstrated that radiomic features extracted from T2-weighted MR images can predict lymph node metastasis in bladder cancer. Another study successfully used radiomic features from CT scans to predict mediastinal lymph node metastasis of lung adenocarcinoma and obtained a promising AUC of 0.97 (22).

Radiomic features can reveal minute changes in the tumor histological anatomy that are difficult to quantitatively identify with the naked eye. In this study, we found that these radiomic features extracted from DCE-MRI of primary tumors are correlated with axillary SLN metastasis and can be used for artificial intelligence model development. This result indicates that these features are characteristic of breast cancer and may reflect the biological tumor behavior. All six features were higher-order statistics features, which were obtained through an image transformation of the original image. These features are indices of intensity and texture features that can better display intratumor heterogeneity and subtle alterations in tissue morphology (23). An image transformation with a filter can eliminate noise in the image or sharpen the image and does not alter the semantic meaning of the features. For example, glcm_Idn is an measure of the local homogeneity of an image, glrlm_GrayLevelNonUniformity (GLNU) can effectively reflect the similarity of gray-level intensity values in the image and glszm_GLNU measures the variability of gray-level intensity values in the image. Logarithm_glszm_GLNU, squareroot_glrlm_GLNU, and logarithm_glrlm_GLNU were all calculated from gray-level intensity features. Thus, these results suggested that the gray-level intensity values might be of greater importance.

DCE-MRI is an effective modality to diagnose breast cancer by evaluating the morphology and hemodynamics of tumors. This method can provide images with high temporal resolution, high spatial resolution and a high signal-to-noise ratio. DCE-MRI has many scanning phases, and currently, there is no consensus on which phase of feature extraction offers the best prediction. Recently, Liu et al. (24) applied radiomic features extracted from the first enhancement phase of primary tumors to predict SLN metastasis, with an AUC of 0.806. In this study, we used the strongest phases of tumor enhancement and obtained a higher accuracy. Compared to the first enhanced phase, delineating the VOIs in the strongest enhanced phase according to the time signal curve showed the lesion boundaries more clearly. Moreover, the strongest enhanced phase can better reflect the tumor's heterogeneity and invasiveness (25). In this study, we did not include the routine T2-weighted imaging and DWI in the data analysis and artificial intelligence model development. Most lesions on T2WI and DWI images show unclear lesion borders, and it is difficult to completely segment the lesions, especially for tumors with severe hyperplasia of the mammary glands. In fact, Yu et al. (26) applied T2-WI and DWI texture features to predict SLN metastasis and obtained relatively low AUC values of 0.770 and 0.787, respectively. DCE-MRI can provide more information about tumor heterogeneity, and DCE-MRI-derived features can better predict SLN metastasis.

In conclusion, in this study, we demonstrated the feasibility of combining artificial intelligence and radiomics from the DCE-MRI of primary tumors to predict SLN metastasis in breast cancer by delineating the VOIs in the strongest enhanced phase. This approach is a non-invasive and highly accurate method for the preoperative prediction of SLN metastasis; it can guide further treatment planning and prevent unnecessary invasive SLNB. The current study also has some limitations. First, we delineated only VOIs in the strongest enhanced phase using the time signal curve. Second, the number of patients enrolled in our study was limited, although the results are promising. In future work, we will further confirm our results with larger and more homogenized samples, and we will analyze all the different enhanced phases of DCE-MR images.

## Data Availability Statement

The datasets generated for this study are available on request to the corresponding author.

## Ethics Statement

The studies involving human participants were reviewed and approved by The Medical Ethics Committee of the Second Affiliated Hospital of Chongqing Medical University. The patients/participants provided their written informed consent to participate in this study.

## Author Contributions

JL, DS, and CLi study design. JL and DS: study conduct. TN: clinical data support. DS, CLiu, and LF: data collection. YX and XZ: data processing. JL, DS, YX, and XZ: statistical data analysis. JL, DS, ZF, WS, and CLi: data interpretation. LC and DG: MRI reading. JL and DS: drafting manuscript. All authors: revising and approving manuscript content. All authors contributed to editing the manuscript, read, and approved the final version.

### Conflict of Interest

The authors declare that the research was conducted in the absence of any commercial or financial relationships that could be construed as a potential conflict of interest.
